# Validation of 3D cryoEM single particle reconstruction correctness and handedness with Ewald’s sphere correction

**DOI:** 10.1101/2024.08.29.610390

**Published:** 2024-08-30

**Authors:** Raquel Bromberg, Yirui Guo, Dominika Borek, Zbyszek Otwinowski

**Affiliations:** a Ligo Analytics, 2707 Chunk Ct., Dallas, TX, 75206, United States; b Department of Biophysics, The University of Texas Southwestern Medical Center, 5323 Harry Hines Blvd., Dallas, TX, 75390, United States; c Department of Biochemistry, The University of Texas Southwestern Medical Center, 5323 Harry Hines Blvd, Dallas, TX, 75390, United States

**Keywords:** Ewald’s sphere correction, complex-valued images, validation, handedness determination, cryogenic electron microscopy single particle reconstruction

## Abstract

The correct description of quantum scattering places the observed scattering contributions on the Ewald’s sphere and its Friedel mate. In electron microscopy, due to the large radius of the Ewald’s sphere, these contributions are typically merged during data analysis. We present an approach that separates and factorizes these contributions into inversion-symmetric and inversion-antisymmetric components. The correlations between reconstructions derived from these symmetric and antisymmetric components enable the automatic determination of handedness and provide additional validation for the quality of 3D reconstructions. These correlations are robust enough to be routinely used in single-particle reconstructions, even at resolutions below the limit where the curvature of the Ewald’s sphere affects the overall signal-to-noise ratio.

## 1. Introduction

**Figure 1:**
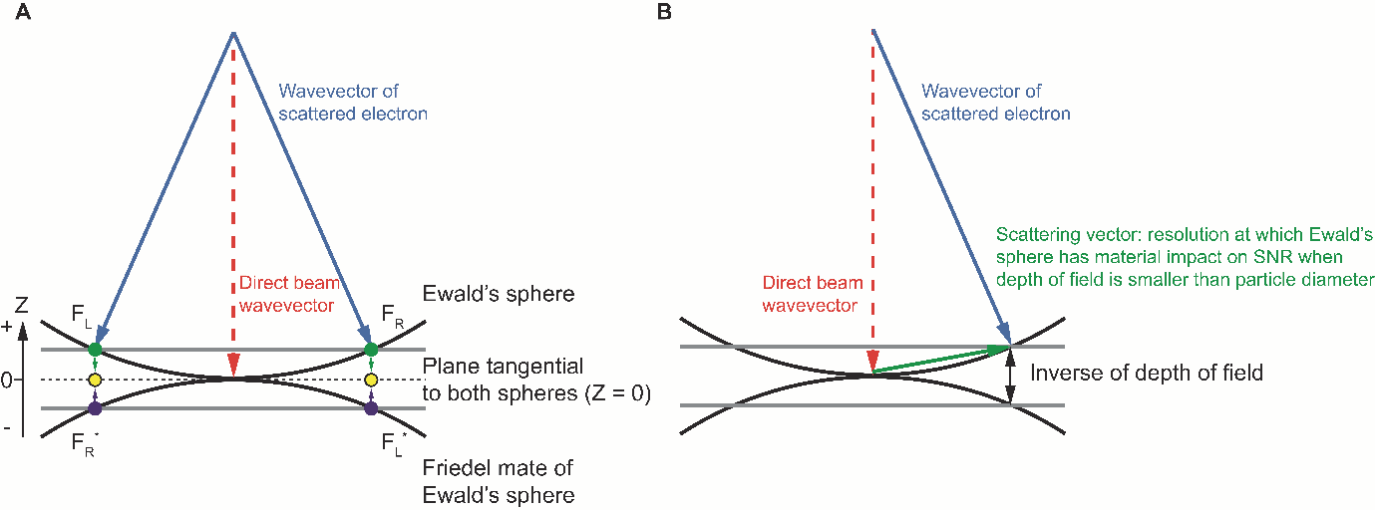
Geometry of diffraction measurement. A) Ewald’s sphere and its Friedel mate. Friedel symmetry in 3D (flip the origin) creates the Friedel mate of the Ewald’s sphere. We project the Fourier transform of the 2D image onto the Ewald’s sphere, and the complex conjugate of this Fourier transform to the Friedel mate of the Ewald’s sphere. B) The Ewald’s sphere is defined by wavevectors of scattered electrons and the separation between the Ewald’s sphere and its Friedel mate defines the inverse of the depth of field where it has material impact on the SNR.

The conservation of energy in elastic scattering is geometrically represented by the Ewald’s sphere (Ewald, 1969). Consequently, in cryogenic electron microscopy single particle reconstruction (cryoEM SPR), the Fourier transform of the phase contrast image represents the data on the Ewald’s sphere and its Friedel mate, which is a second, inverted copy of the Ewald’s sphere, representing the complex conjugate of the wave function also contributing to measurement. Due to the very short wavelength of electrons, the Ewald’s sphere is almost flat at the resolutions of interest, and so contributions from data projected on the Ewald’s sphere and its Friedel mate can be merged on the plane that is tangential to both spheres (Figure 1A dashed line, Z = 0, Z is a reciprocal space coordinate with respect to beam direction, not to be confused with defocus z). For this reason, 3D reconstruction performed in reciprocal space starts from only considering information merged on the plane (the planar approximation) (Figure 1A, yellow dots) (Cohen *et al.*, 1984, Derosier & Klug, 1968, Glaeser, 2019). However, more precise description of the projected data using a curved Ewald’s sphere can improve 3D reconstruction (Figure 1A, green and blue dots). Apart from the occasional improvements in resolution (Gureyev *et al.*, 2022, Zhu *et al.*, 2018, Tan *et al.*, 2018, Russo & Henderson, 2018, Leong *et al.*, 2010, Wolf *et al.*, 2006, DeRosier, 2000), we have identified additional benefits of considering Ewald’s sphere curvature, which we discuss here.

In a cryoEM SPR experiment, the major source of contrast in a TEM image comes from interference between direct and elastically scattered electrons. A scattered electron has a complex value wave function, $F_{e}$, while $F_{d}$ represents the wave function of the unscattered direct beam (Figure 1B). By the quantum mechanics Born postulate, the measured phase contrast signal is proportional to:

(Eq.1) $$\left(F_{e}+F_{d}\right){\left(F_{e}+F_{d}\right)}^{*}$$

where ${\left(F_{e}+F_{d}\right)}^{*}$ is the complex conjugate of $\left(F_{e}+F_{d}\right)$. In the approximation that ignores Ewald’s sphere curvature (e.g., at low resolution), the observed phase contrast signal (aberration corrected image in real space) results only from the real part of the wave function $F_{e}$ in real space. In the planar approximation, the contribution from the imaginary component of the aberration-corrected image cancels out in reconstruction space. At high resolution, contributions from both the real and imaginary parts of the wave function $F_{e}$ affect the reconstruction, and that is when Ewald’s sphere curvature should be considered.

Including Ewald’s sphere curvature in cryoEM SPR improves the signal-to-noise ratio (SNR). The magnitude of improvement depends on the ratio of the particle size (particle diameter) to the depth of field at a given resolution (Figure 1B). This ratio increases rapidly with the resolution of scattering. Depth of field L is given by:

(Eq. 2) $$L=\frac{2d^{2}}{\lambda }$$

where d is resolution in Å, and λ is electron wavelength (DeRosier, 2000, Downing & Glaeser, 2018, Glaeser, 2019). When the depth of field is smaller than particle diameter, Ewald’s sphere curvature should be considered in reconstruction. Conversely, if at the limiting resolution the depth of field is larger than particle diameter, consideration of Ewald’s sphere curvature adds little to the SNR and its proxy, the Fourier Shell Correlation (FSC) between half-maps (half-maps FSC).

Considering Ewald sphere curvature also provides other benefits besides increasing the SNR. It was postulated that the imaginary part of the wave function $F_{e}$ can provide handedness differentiation (Wolf *et al.*, 2006). However, the proposal was not implemented at that time and doubts were raised recently on (Heymann, 2023) whether handedness determination with this approach is practical. We created a sensitive handedness determination method by signal factorization and show that automatic handedness determination that considers the imaginary component of the wave function $F_{e}$ can be used for a broad range of projects. We also show that this method can be used for validation of 3D reconstruction independently of other statistical indicators such as half-maps FSC (Sousa & Grigorieff, 2007, Egelman, 2024a, Subramaniam *et al.*, 2016).

### 1.1 Algebra of reconstruction based on the Ewald’s sphere

Data acquisition happens on 2D detectors, and the first level of description is in the detector (2D) space. The consideration of Ewald’s sphere curvature requires expanding the description to three-dimensional geometry.

We start with the exit wave function $F_{e}=e^{i\varphi }$ using φ=2πW, where W is the wavefront function. In the first approximation, ignoring higher-order aberrations, the wavefront function is:

(Eq. 3) $$W\left(k\right)=-\frac{z\lambda k^{2}}{2}$$

where z is the defocus, and k is the scattering vector. We apply our CTF based estimate of the wavefront function as a phase shift to the observed image to generate the phase shifted image (Bromberg *et al.*, 2023). The wavefront function can also be split into base $\left(z\right)$ and correction $\left(\Delta z\right)$, where Δz represent the defocus difference in the correction to the wavefront function.

In reciprocal (momentum) space, the exit wave function has both Hermite and anti-Hermite components which by the Fourier transform are equivalent to the real and imaginary parts in real space. Experimentally, we measure interference between scattered and direct beams. This measurement contains a mixture of signals from real and imaginary components, because scattering in two opposing directions (Figure 1A, F_R_ and F_L_) creates the same periodicity (DeRosier, 2000). Scattering waves in opposite directions (F_R_ and F_L_) are far apart in the Fourier transform of the image (Fig. 1A, F_R_ and F_L_ represented by green dots). When mapped on the Ewald’s sphere and its Friedel mate, they become close (Figure 1A, F_R_ and F_L_*, or F_L_ and F_R_*, represented by green and blue dots), with their distance resulting from the contribution of the Ewald’s sphere curvature.

## 2. Methods

### 2.1 Factorization of handedness signal

**Figure 2:**
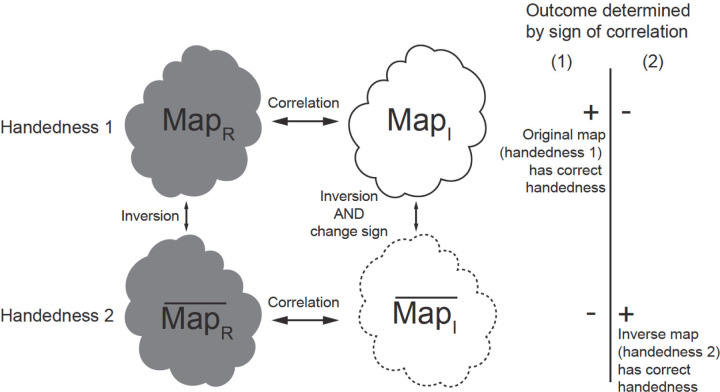
Separate reconstructions from the real component and imaginary component help handedness determination. $Map_{R}$ and $\bar{Map_{R}}$ are related by inversion only. $Map_{I}$ and $\bar{Map_{I}}$ are related by inversion and flipped sign. FSC between $Map_{R}$ and $Map_{I}$ and FSC between $\bar{Map_{R}}$ and $\bar{Map_{I}}$ are used in handedness determination. If $Map_{R}$ has the correct handedness as in situation (1), the FSC between $Map_{R}$ and $Map_{I}$ will be positive; if $\bar{Map_{R}}$ has the correct handedness as in situation (2), and FSC between $\bar{Map_{R}}$ and $\bar{Map_{I}}$ will be positive.

We noticed that the Hermite and anti-Hermite components of the wave function, or equivalently in real space its real and imaginary parts, have a different relationship to the inversion of a 3D reconstruction. Map statistics (including histogram and half-maps FSC) from the reconstruction using the Hermite (real) component of the wave function $\left(Map_{R}\right)$ are identical to map statistics from the inverted reconstruction ($\bar{Map_{R}}$, inversion can be achieved by resetting particle orientation from Euler angles α, β, γ to α, β, γ+180°, where γ represents rotation in the image plane) (Figure 2). For this reason, handedness cannot be derived from inspection of $Map_{R}$ and $\bar{Map_{R}}$ alone. However, the reconstruction from the anti-Hermite (imaginary) component of the wave function $\left(Map_{I}\right)$ also changes sign upon inversion $\left(\bar{Map_{I}}\right)$ (Figure 2). This allows us to factorize the problem of handedness determination. We can separately perform reconstructions from the real component and imaginary components and investigate the sign of correlation between these two reconstructions (correlation between $Map_{R}$ and $Map_{I}$, and correlation between $\bar{Map_{R}}$ and $\bar{Map_{I}}$) (Figure 2). The correct handedness will result in positive correlation, while the wrong handedness will lead to negative correlation. These correlations are informative even if the resolution is moderate because we typically have many particle images, so weak signal becomes significant when it is averaged over all of them. Typically, we have enough particles to calculate this correlation in resolution shells, so here we present it as FSC between two reconstructions from the same data: one based on the real component and other based on the imaginary component (FSC between $Map_{R}$ and $Map_{I}$, and FSC between $\bar{Map_{R}}$ and $\bar{Map_{I}}$). If $Map_{R}$ has the correct handedness, the FSC between $Map_{R}$ and $Map_{I}$ will be positive; otherwise $\bar{Map_{R}}$ has the correct handedness, and FSC between $\bar{Map_{R}}$ and $\bar{Map_{I}}$ will be positive (Figure 2). When FSCs for both possibilities are plotted, the mirror feature of the FSC plot makes it very intuitive to assess if the handedness signal is significant. In principle, the highest sensitivity will be obtained by averaging correlations across resolutions, but typically even a single shell is highly statistically significant.

Because of the relationship between real and imaginary components-based reconstructions towards the 3D map and its inversion, the inverted map can be derived automatically from the original map, so we do not double the computation time. It is also worth noting that here we assume a standard approach for the reconstruction so there is no particle orientation bias from using a reference that prefers one handedness over the other, i.e. the imaginary component is not used in reference until the decision about chirality of the solution is made.

### 2.2 Resolution considerations

When we observe a particle at some defocus setting, we can consider the actual defocus value at each z-plane within the particle, with its +/− range being defined by particle radius. In a reconstruction that has all possible projections, the average defocus spread can be characterized by twice the molecular radius of gyration, $R_{g}$. When the wavefront change caused by z-depth variations within the particle reaches the value of ~0.5 ($\Delta W\left(k\right)$, defined in Eq. 3 as being in the range of +/− 0.25), considerations of defocus variation dominate the reconstruction and including Ewald’s sphere curvature becomes necessary. This happens at resolution higher than $\sqrt{2R_{g}\lambda }$. Such resolutions are achievable for some larger, rigid molecules, but are not typical. For example, virus AAV2 has a radius of gyration of 120 Å. The resolution at which the defocus starts to dominate is equal to ~2.2 Å (Tan *et al.*, 2018), assuming 300 kV electrons with wavelength of 0.019687 Å. The resolution at which the defocus determination starts to dominate in the case of apoferritin, which has a radius of gyration of 60 Å, is 1.55 Å for 300 kV electrons. These cases are examples of where Ewald’s sphere curvature is a critical contributor during the final steps of analysis, but in the case of most others, we are well below the resolution limit where defocus variation (i.e. Ewald’s sphere curvature) is material for the results. Our goal is to obtain reliable handedness determination below the very high resolutions discussed above and we achieved it by factorization of the signal into real and imaginary parts and performing separate reconstructions with them.

### 2.3 Implementation

**Figure 3:**
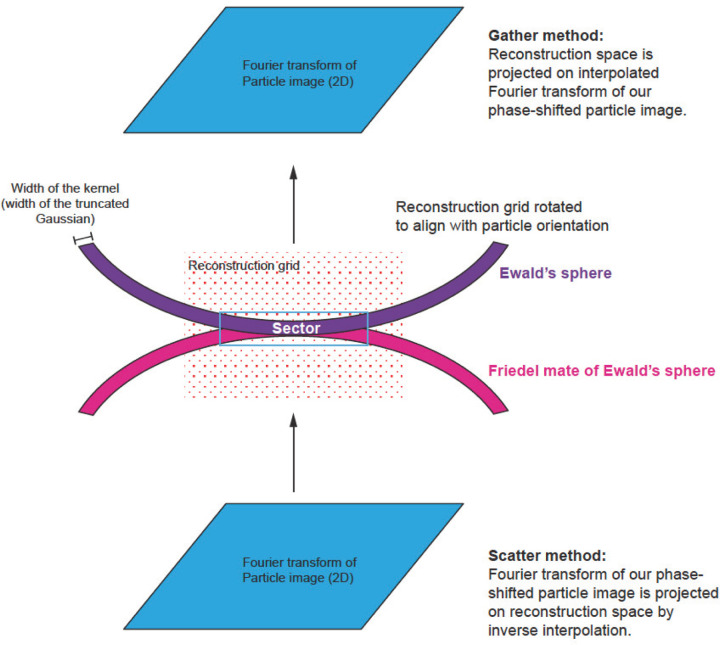
3D geometry of reconstruction from a single planar image. There are two methods that are used in the field: the scatter method and the gather method. The scatter method takes the Fourier transform and for each Fourier element, assigns the signal and its weights to the nearest points on the 3D reconstruction grid with linear interpolation weights. However, the calculation is not linear interpolation but rather the inverse of it. This is why the method is called ‘scatter’. The gather method starts by mapping each point from the reconstruction grid onto the Fourier transform of the image and then calculates an interpolated value of the image and assigns it to a single 3D reconstruction grid point. With Ewald’s sphere correction, both methods have to take into account the need to follow the surface of a sphere rather than a plane, and to consider the additional weight associated with the distance of a reconstruction grid point from the Ewald’s sphere. This additional weight may follow various interpolation kernels, e.g. linear, Kaisser-Bessel, Gaussian, etc.

The numerical calculations associated with reconstructions are performed on grids in Fourier space. The grid associated with the source signal is a 2D Fourier transform of a boxed particle image corrected for CTF. The grid associated with the reconstruction is a 3D Fourier transform of two sums from 2D contributions. One sum consists of their weighted signal, and the other sum is of the weight squared. The weight originated from the product of two terms: one is associated with an interpolating kernel (Gaussian kernel in our case), the other is associated with the interference term between the Ewald’s sphere and its Friedel mate. The interference term corresponds to CTF in a planar approximation for real signal. The particle orientation is quite random with respect to the 3D grid, so the grid points associated with the 2D particle information fall between the grid points of the 3D reconstruction. Therefore, we need to define a method to transfer information from one grid to another. There are multiple schemes that bridge the gap between the two grids, and they all involve some type of interpolation/integration kernel, e.g., the Kaiser-Bessel Window function (Abrishami *et al.*, 2015, Strelak *et al.*, 2019). The reconstruction calculations are performed in reciprocal space, and can start from traversing the Fourier transform of the particles (data space) and then mapping data space grid points on reconstruction space —such methods are called scatter-based (each data space grid point can be scattered on eight or more reconstruction grid points), or starting from traversing the reconstruction grid point and then projecting it to data space—such methods are called gather-based (Strelak *et al.*, 2019) (Figure 3). Both types of methods are used, and they perform equivalently in terms of accuracy, but may have different speed of calculations depending on the implementation. We present the details of our approach using a gather-based method with a Gaussian kernel.

We extend the gather method from Strelak *et al.* where only planar approximation was considered, to include contributions from both the Ewald’s sphere and its Friedel mate (Eq. 1 in (Strelak *et al.*, 2019)):

(Eq. 4) $$F_{3D}\left(\bar{R}\right)=\int {\hat{F}}_{3D}\left(\bar{Q}\right)K\left(\bar{R}-\bar{Q}\right)d\bar{Q}$$

where $\bar{R}$ is a coordinate within a 3D reconstruction grid, $\bar{Q}$ is a kernel convolution integration variable, and K is the interpolation kernel expanded to consider interference between the two spheres. ${\hat{F}}_{3D}\left(\bar{Q}\right)$ are experimental data mapped to both spheres.

We first Fourier transform each 2D particle image collected by the detector and apply CTF as a phase-shift term. We then calculate Hermite (real) and anti-Hermite (imaginary) components of the particle. Next, we accumulate separately the reconstruction signal from real and imaginary components. While the orientation of particles is the same for both reconstructions, the contribution from particle images (real vs. imaginary) and weights are very different both in the CTF term and in how the kernel contributions are summed from both spheres.

**Figure 4:**
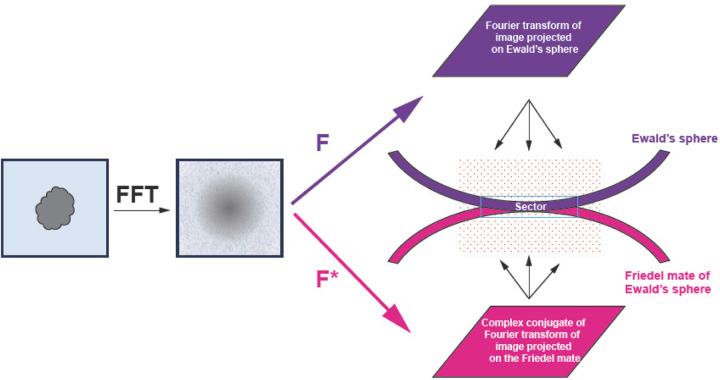
Geometry of 3D reconstruction. From an image, we calculate the Fourier transform and the complex conjugate of it. The Fourier transform (F) is mapped on the Ewald sphere while the complex conjugate (F*) is mapped on the Friedel mate of the Ewald’s sphere. This calculation is performed on the reconstruction grid where both spheres may contribute to the same grid point and these contributions add as complex structure factors. When these two spheres are almost touching, this addition generates a modulation known as Thon rings. Under this condition, only the real component of the image contributes to the reconstruction. Note the orientation of the 3D reconstruction grid relative to Ewald spheres is different for each particle.

For each particle, we identify a relevant 3D sector for reconstruction that is thick enough to account for a Gaussian kernel and Ewald’s sphere curvature (Figure 3 and Figure 4). We map each point of the sector onto our particle’s data space.

In the gather method, the kernel defines the weight contribution from the Fourier transform of the phase-shifted detector image mapped onto the Ewald’s sphere. The weight factor is the sum of squares of the summed kernel contributions from the Ewald’s sphere and its Friedel mate.

**Figure 5:**
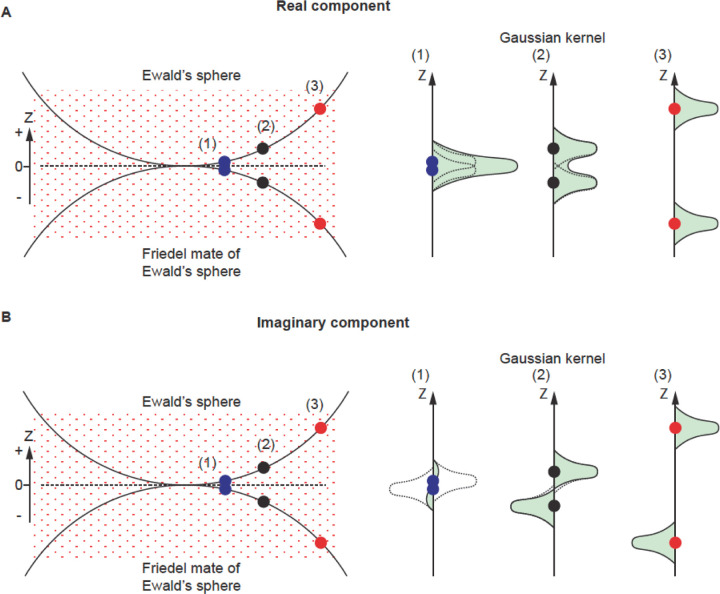
Gaussian kernel contributions for real and imaginary component. A) For the real component, the kernel contributions from the Ewald’s sphere and its Friedel mate are always positive regardless of their distance in Z (right panel (1)-(3), each kernel is represented by a dashed curve), so they contribute to the weight factor in an additive way (solid curve with green area). B) For the imaginary component, the kernel contributions from the two spheres have the same shape (Gaussian) but of opposite signs (right panel (1)-(3) dashed curve). When the reconstruction space point is close to Z = 0 such as in (1) (blue dots), the two kernels will cancel out each other so that the sum of the kernel contribution at such a point is close to 0. As a result, the contribution to the weight factor (square of the summed kernel contribution) at such a point is small. For points away from Z = 0, as in (2) and (3) (blue and red dots), each sphere’s kernel contribution stops overlapping with the other and the sum of the weight squared increases.

For the real component, the kernel contributions from the Ewald’s sphere and its Friedel mate are always positive regardless of their distance in Z (Figure 5A, right panel (1)-(3), each kernel is represented by a dashed curve), so they contribute to the weight factor in an additive way (Figure 5A, right panel (1)-(3) solid curve with green area). The SNR is calculated from these sums by an established method (Penczek, 2002, Scheres & Carazo, 2009, Sigworth *et al.*, 2010, Zivanov *et al.*, 2018).

For the imaginary component, the kernel contributions from the two spheres have the same shape (Gaussian) but of opposite signs (Figure 5B, right panel (1)-(3) dashed curve). When the reconstruction space point is close to Z = 0 such as in Figure 5B (1), the two kernels will cancel each other out so that the sum of the kernel contribution at such a point is close to 0. As a result, the contribution to the weight factor (square of the summed kernel contribution) at a such point is small. This is the reason why we do not have imaginary signal contribution when using the planar approximation for reconstruction (e.g., for low resolution reconstruction). However, the sum of weight squared increases when the points move away from Z = 0, where each sphere’s kernel contribution stops overlapping with the other (Figure 5B, (2) and (3)). Similar to the treatment of the real component, the sum of the weight factor squared is the source of our SNR calculation for the imaginary component reconstruction. For this reason, the SNR for the imaginary component reconstruction is low at low resolution and improves when resolution increases, until the noise is too high. One should note that Thon rings originate from the real image component in the planar approximation. When considering the Ewald’s sphere curvature, the real component’s dominance is responsible for Thon ring modulation. This modulation disappears at higher resolution when the imaginary component’s contribution becomes equally strong. As a result, the maximum resolution of Thon rings is not the resolution limit of the reconstruction. The resolution limit of reconstruction could either be higher or lower than the Thon ring maximum resolution.

### 2.4 Weighting of the correlation between real and imaginary reconstructions

In calculations of the correlation between reconstructions from real and imaginary components, we include an additional factor to consider how the particle size and depth of field affect the reconstruction. The reconstruction from the imaginary component uses only out-of-focus signal (when the reconstruction point is close to Z = 0 or the in-focus, imaginary component vanishes). For typical resolution when the particle diameter is smaller than the depth of field, the single particle reconstruction from the imaginary component is modulated by ${\left(\Delta z\right)}^{2}$, where Δz is the coordinate difference between a slice of the particle to the centre of the particle in the beam direction in real space (same as the defocus difference for the correction to the wavefront function at that slice). As a result, assuming isotropic distribution of particle orientations, the overall reconstruction based on the imaginary component $\left(Map_{I}\right)$ will be modulated by $R^{2}$, where R is the distance between a point in the 3D reconstruction map and the centre of this map. The reconstruction from the real component does not have this modulation, and so to make the two reconstructions more similar, we applied the $R^{2}$ modulation to the result from the reconstruction based on the real component $\left(Map_{R}\right)$. The $R^{2}$ scaling is invariant with respect to the inversion of the reconstruction $\left(\bar{Map_{R}}\right)$, so it cannot change the sign of correlation between two reconstructions). The FSC curve representing the correlation between the reconstruction from the imaginary component and the $R^{2}$-modulated real component is the main result of our method. We refer it to as I-R FSC.

### 2.5 Expected magnitude of imaginary component signal

Reconstructions from the imaginary and real components describe the same object, so they are always correlated. However, the magnitude of the correlation can vary between experiments. If we could make a prediction for the resolution-dependence of this correlation (predicted I-R FSC, based on SNR from half-maps FSC), then we would obtain a validation criterion for the correctness of our reconstruction by comparing the I-R FSC and the predicted I-R FSC. This validation was successfully applied to correct reconstructions, even when the resolution was intermediate (2.5 – 4 Å). We also performed negative tests on a dataset that based on half-maps FSC seemed promising, but didn’t result in sensible reconstruction maps. In this case, the I-R FSC show variable signs and low amplitudes consistent with noise and is not correlated well with the predicted I-R FSC (see more details in the Results section).

This type of validation is inherently qualitative rather than quantitative, as the amount of structural disorder may depend on the distance from the centre of the molecule and this disorder would attenuate the expected correlation. Also, preferred orientation would perturb this correlation by perturbing the randomness of ${\left(\Delta z\right)}^{2}$ contributions to the reconstruction 3D map, so subsequent considerations are based on simplifying assumptions: 1) protein is roughly spherical, 2) random orientation of particles, 3) particles have no correlation of disorder with the distance from centre of the particle, and 4) randomness of the defocus error.

### 2.6 Consideration of kernel width

The starting point for the expected signal (SNR) from the imaginary component can be derived from either real or reciprocal space considerations. In real space, we can integrate the signal that appears on each pair of +z and −z planes (e.g., planes on the opposite side of the centre plane of the particle in the beam direction) that increases initially with $\Delta z^{2}$. In reciprocal space, we can calculate the second moment of the difference between two Gaussian kernels (one from the Ewald’s sphere and the other from its Friedel mate), but we have to consider that for this purpose, we should use a Gaussian kernel width that corresponds to the particle’s moment of inertia, e.g., for a small moment of inertia, we have small kernel width in real space that corresponds to wide kernel width in reciprocal space. However, in a typical reconstruction, which also applies to our real component only reconstruction, we use a narrower Gaussian kernel in reciprocal space to minimize artifacts of multiplication by this kernel in real space. To simplify implementation, we also use a narrower kernel for SNR calculation and adjust the result by a compensating factor which also accounts for other departures from our oversimplified SNR theory and assumptions about the particles discussed in the above session. For presentation purposes, we convert the predicted SNR calculated for the real and imaginary components into a predicted FSC between the real and imaginary component reconstructions (Predicted I-R FSC) (Penczek, 2002).

## 3. Results

### 3.1 Handedness determination features

**Figure 6:**
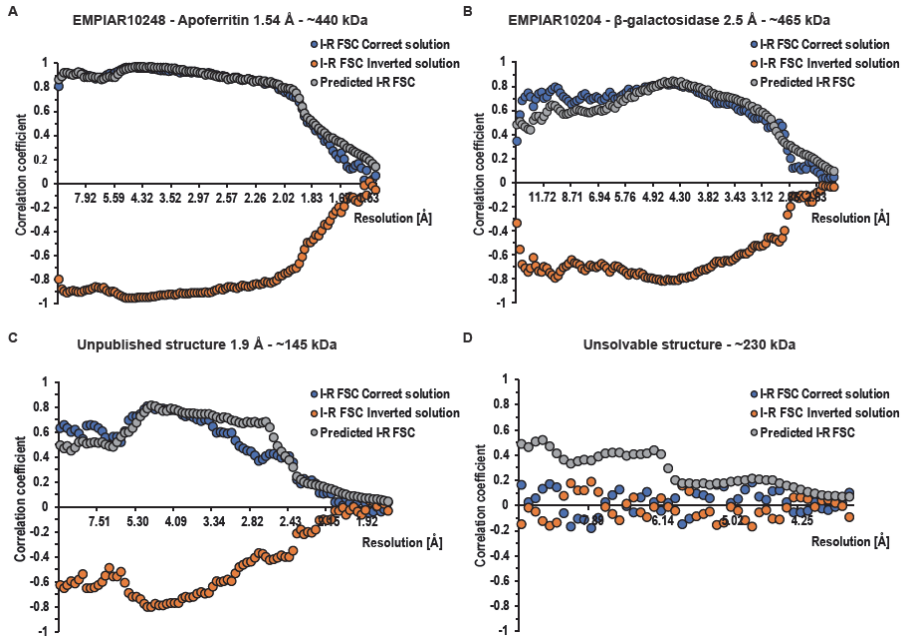
FSC statistics from the four examples. The blue and orange curves represent the observed correlations between reconstructions calculates from the imaginary and real signals (I-R FSC). There are two curves which represent the correct solution (blue, I-R FSC Correct solution) and inverted solution (orange, I-R FSC Inverted solution). Due to factorization of the problem, these two curves are mirror images of each other. Separation between these curves indicates the significance of the handedness determination problem. For panels A-C, even a single resolution shell would provide more than sufficient statistical signal to indicate which solution is correct. The grey curve represents idealized predictions for the correlation when the 3D solution is right (Predicted I-R FSC). A validation criterion for the correctness of the reconstruction can be obtained by comparing the I-R FSC (blue) and the predicted I-R FSC (grey). This prediction may help to interpret the lack of separation between the blue and orange curves, to be attributed to the incorrectness of the solution (panel D) rather than the lack of statistical signal.

For each structure determination, the core result is an FSC plot between real-based and imaginary-based reconstructions (I-R FSC). For well-ordered, large molecules, the FSC can reach the level of 0.97 (Figure 6A, blue curve). Interestingly, this level is reached not at the highest resolution where the signal ratio between imaginary and real reconstruction has the largest relative magnitude, but rather at intermediate resolution (~5 Å) where the measurement accuracy is high, which compensates for the moderate signal ratio between imaginary and real reconstructions. The significant FSC continues down to at least 10 Å resolution. In other projects, the FSC has reached various values, with the maximum being typically at 4–6 Å resolution (Figure 6BC). The absolute configuration was unambiguously determined even if the limiting resolution was around 4 Å. We assume a normal procedure where reconstructions are based on particle orientation (i.e. pose) refined against a 3D map which ignores Ewald’s sphere considerations, or alternatively using reconstruction based on the real component of the image. Under such conditions, we do not expect any model bias in the imaginary-based reconstruction.

### 3.2 Validation based on expected vs observed signal

For validation purposes, we investigated the relationship of the I-R FSC curves of the correct solution (Figure 6, blue curve) to the predicted I-R FSC (Figure 6, grey curve). We do not expect perfect agreement between those curves because molecules may not be globular and/or may have disorder correlated with distance from the molecule centre, defocus determination during reconstruction may have systematic errors, and there may be preferred orientation, etc. Overall, we found good qualitative agreement indicating that the signal from the imaginary part is properly analysed and does not have major artifacts. This is important because SNR from the imaginary component has a lower value in general and dominates the I-R FSC. We also looked at maps of the imaginary component reconstruction for cases where the signal was very strong. The imaginary maps had the expected features of higher values at larger distances from the reconstruction centre and were missing the low resolution contribution.

### 3.3 Examples

#### 3.3.1 Ideal case: apoferritin

We reconstructed apoferritin from EMPIAR-10248 (Kato *et al.*, 2019) to 1.54 Å with O symmetry. All the core assumptions were satisfied for the case of apoferritin: the molecules are globular, preferred orientation is almost isotropic due to 24-fold symmetry, particles were of a single structural class, and the number of planes contributing to the reconstruction was very large. The I-R FSC curve went up to ~0.97 at about 5 Å resolution and agreed very well with the predicted I-R FSC (Figure 6A).

#### 3.3.2 Molecule of anisotropic shape: β-galactosidase

We reconstructed the β-galactosidase dataset (EMPIAR-10204) to 2.5 Å in D2 symmetry. This molecule has an anisotropic moment of inertia with an ellipsoid shape. This may explain why the predicted I-R FSC curve had some systematic mismatch with the I-R FSC depending on resolution. The resolution was about 2.5 Å with about 200,000 contributing planes, which is not a large number for a molecule with D2 symmetry (Figure 6B).

#### 3.3.3 Small globular protein with strong preferred orientation (unpublished data)

We applied our method to a small globular protein (145 kDa) dataset reconstructed to high resolution (1.9 Å). The protein showed strong preferred orientation and the reconstruction was done imposing a C5 symmetry. The I-R FSC curve matches the predicted I-R FSC, albeit imperfectly, most likely due to preferred orientation. The small molecule size may create some systematic errors with defocus determination per particle, which are of no significance to the real reconstruction, but may affect the imaginary signal reconstruction (Figure 6C).

#### 3.3.4 Dataset with uninterpretable map albeit promising half-maps FSC with intermediate resolution

We also analysed a problematic case that did not converge to a proper solution in standard reconstruction, although the half-maps FSC overestimated the resolution to 3.8 Å (Figure 6D). The I-R FSC correlation did not show any signal, supporting the expected lack of correlation between the contribution from the real part and the contribution from the imaginary part. The expectation for a properly resolved structure (predicted I-R FSC) was above the noise level.

## 4 Discussion

We analysed the consequences of performing separate reconstructions with the real and imaginary components of the scattered (exit) electron wave. The mathematical rule is that the reconstruction from the real component has the same quality for the solution $\left(Map_{R}\right)$ and the inverted solution $\left(\bar{Map_{R}}\right)$, i.e. these two solutions are exactly related by inversion. However, for the reconstruction from the imaginary component, the solution $\left(Map_{I}\right)$ and the inverted solution $\left(\bar{Map_{I}}\right)$ have opposite signs. The sign of the correlation between the real and imaginary reconstructions unambiguously determines whether the solution or its inverse is correct. This correlation is strong even for typical cases in cryoEM SPR when the reconstruction resolution is limited to ~3–4 Å, or when particles are not too large (diameter <100 Å). Additionally, this correlation presents a new type of validation criterion. Since typical refinement schemes do not use the imaginary component of the scattered wave, there is no refinement bias towards this component, and the correlation between the imaginary component-based reconstruction and the real component-based reconstruction can only arise if the solution is correct.

Considering Ewald’s sphere curvature makes calculations more exact, but also slower and more complex. Using the traditional planar approximation is sensible, particularly for the earlier steps of reconstruction, even for high resolution or large particles. The I-R FSC presented here could be the final step for medium to low resolution structures, followed by inversion of the structure if so indicated (e.g., when the handedness of the original map is wrong). For high resolution structures, the appropriate inversion needs to be performed first, followed by reconstruction with Ewald’s sphere curvature calculations. It is important to use well-characterized handedness of micrograph images, as handedness violating transformations (e.g., flips along the axis) can happen in some workflows (e.g., during conversion of image formats). Other than for this, the method is reliable and does not need additional validation of handedness.

In terms of validation, the proposed method is not a replacement for a half-maps FSC. However, it is interesting from the theoretical perspective that validation can be achieved without half-maps. Our method would provide much stronger signal (and so validation) for data collected at low energy, for example with a 100 kV electron beam, where analysis following Ewald’s sphere curvature would be indicative of the reconstruction correctness at a much lower resolution. This method may also have practical utility as an indicator of whether a project is on the right or wrong track. There are data discussed in the literature (Egelman, 2024b) where such validation (I-R FSC) would provide a definitive answer.

## Synopsis

The implementation of Ewald’s sphere correction that separates the chirality-sensitive component provides handedness determination and validation of 3D reconstruction.
