# Supplementary material for: Validation of 3D cryoEM single particle reconstruction correctness and handedness with Ewald’s sphere correction
Source: bioRxiv. 2025 Jul 26:2024.08.29.610390. Originally published 2024 Aug 30. Preprint. [Version 2] doi: 10.1101/2024.08.29.610390 (PMC11383999; doi:10.1101/2024.08.29.610390)
Supplement: Supplement 1 [file NIHPP2024.08.29.610390v2-supplement-1.pdf]

bioRxiv preprint doi: https://doi.org/10.1101/2024.08.29.610390; this version posted July 26, 2025. The copyright holder for this preprint (which was not certified by peer review) is the author/funder, who has granted bioRxiv a license to display the preprint in perpetuity. It is made available under aCC-BY 4.0 International license.


## Supplementary Figure

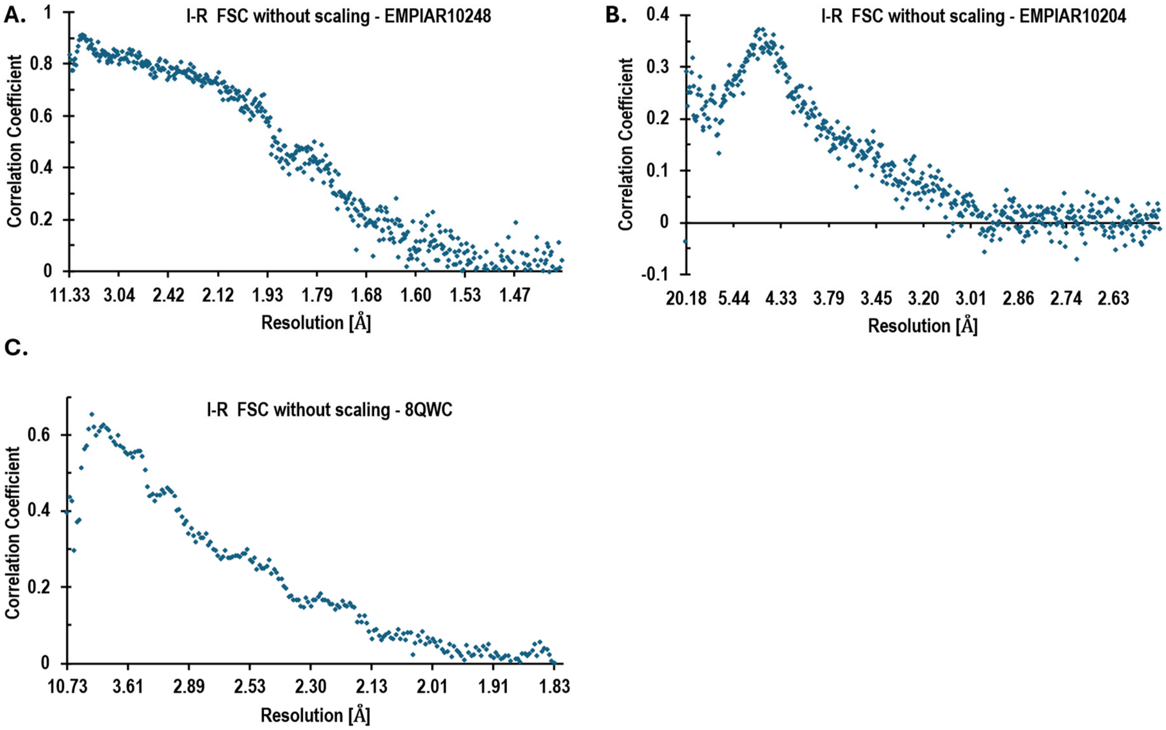


FIG. S1. I–R FSC curves calculated on data not filtered by the Wiener filter. As expected, the correlations between I and R maps are lower for unfiltered data.
